# Hypoimmunogenic human iPSCs expressing HLA-G, PD-L1, and PD-L2 evade innate and adaptive immunity

**DOI:** 10.1186/s13287-024-03810-4

**Published:** 2024-07-02

**Authors:** Norihiro Tsuneyoshi, Tomonori Hosoya, Yuriko Takeno, Kodai Saitoh, Hidetaka Murai, Naoki Amimoto, Rie Tatsumi, Sono Watanabe, Yudai Hasegawa, Eri Kikkawa, Kumiko Goto, Fusako Nishigaki, Kouichi Tamura, Hironobu Kimura

**Affiliations:** HEALIOS K.K. Kobe Research Institute, Kobe KIMEC Center Bldg. 3F, 1-5-2 Minatojima-Minamimachi, Chuo-Ku, Kobe, Hyogo 650-0047 Japan

**Keywords:** Human iPSC, Gene engineering, Hypoimmunogenic pluripotent cells, HLA-G, PD-L1, PD-L2, Suicide gene

## Abstract

**Background:**

The human induced pluripotent stem cells (hiPSCs) can generate all the cells composing the human body, theoretically. Therefore, hiPSCs are thought to be a candidate source of stem cells for regenerative medicine. The major challenge of allogeneic hiPSC-derived cell products is their immunogenicity. The hypoimmunogenic cell strategy is allogenic cell therapy without using immune suppressants. Advances in gene engineering technology now permit the generation of hypoimmunogenic cells to avoid allogeneic immune rejection. In this study, we generated a hypoimmunogenic hiPSC (HyPSC) clone that had diminished expression of human leukocyte antigen (HLA) class Ia and class II and expressed immune checkpoint molecules and a safety switch.

**Methods:**

First, we generated HLA class Ia and class II double knockout (HLA class Ia/II DKO) hiPSCs. Then, a HyPSC clone was generated by introducing exogenous *β-2-microglobulin* (*B2M*), *HLA-G*, *PD-L1*, and *PD-L2* genes, and the *Rapamycin-activated Caspase 9* (*RapaCasp9*)-based suicide gene as a safety switch into the HLA class Ia/II DKO hiPSCs. The characteristics and immunogenicity of the HyPSCs and their derivatives were analyzed.

**Results:**

We found that the expression of HLA-G on the cell surface can be enhanced by introducing the exogenous *HLA-G* gene along with *B2M* gene into HLA class Ia/II DKO hiPSCs. The HyPSCs retained a normal karyotype and had the characteristics of pluripotent stem cells. Moreover, the HyPSCs could differentiate into cells of all three germ layer lineages including CD45^+^ hematopoietic progenitor cells (HPCs), functional endothelial cells, and hepatocytes. The HyPSCs-derived HPCs exhibited the ability to evade innate and adaptive immunity. Further, we demonstrated that RapaCasp9 could be used as a safety switch in vitro and in vivo.

**Conclusion:**

The HLA class Ia/II DKO hiPSCs armed with HLA-G, PD-L1, PD-L2, and RapaCasp9 molecules are a potential source of stem cells for allogeneic transplantation.

**Supplementary Information:**

The online version contains supplementary material available at 10.1186/s13287-024-03810-4.

## Background

Compared to autologous cell therapy, allogeneic therapy can provide “off-the-shelf” readily available products for transplantation and can potentially offer a more cost-effective approach to cell therapy. One major hurdle for allogeneic cell therapy is donor cell rejection by host immune cells triggered by the mismatch of human leukocyte antigen (HLA), which is highly polymorphic. Multiple solutions have been proposed to overcome the hurdle, including transplantation of HLA-matched allogeneic induced pluripotent stem cells (iPSC)-derived cells [1], transplantation of allogeneic cells with the administration of an immunosuppressant [2–5], and development of genetically engineered hypoimmunogenic cells [6].

A common feature of the hypoimmunogenic cell concept is resistance to elimination by the host’s innate and adaptive immune cells. Various strategies for genetically engineering hypoimmunogenic cells have been proposed such as down-regulation of cell surface HLA molecules coupled with exogenous expression of immunosuppressive genes by genetic engineering in pluripotent stem cells [7–11], and forced expression of eight immunomodulatory transgenes in mouse embryonic stem cells [12]. The reduction of HLA class I and II molecules from the cell surface or forced expression of checkpoint inhibitor molecules prevents target killing or the inflammatory T cell response. However, deletion of HLA class I molecules leads to target cell lysis by NK cells due to the missing-self response [13]. The forced expression of non-classical HLA class I molecules, such as HLA-E or HLA-G, has been utilized to prevent NK cell-mediated lysis [7, 9]. CD47 has been reported to inhibit not only phagocytosis by macrophages but also NK cell-mediated lysis [8, 14]. In addition to the add-on strategy, deleting ligands of active NK receptors works to prevent NK cell-mediated lysis [15, 16].

The host immune escape of hypoimmunogenic cells carries a risk of tumorigenicity and treatment-related toxicities. To mitigate this risk, the introduction of a safety switch has been considered. The herpes simplex virus thymidine kinase (HSV-TK) is utilized as a safety switch to control cell growth by Ganciclovir in hypoimmunogenic cells [7, 17]. Another safety switch composed of human genes has been developed. Caspase-9 is a key molecule for activating the apoptosis pathway through caspase-3 and caspase-7 [18]. Dimer formation of caspase-9 triggers the activation of the apoptosis cascade [19]. Applying this characteristic, researchers have developed the catalytic domain of caspase-9 fused with the drug binding domain to induce apoptosis in the presence of the chemical inducers of dimerization (CIDs) [20]. Rapamycin acts as an inhibitor of Mechanistic Target of Rapamycin (mTOR) by binding to the FK506 binding protein 12 (FKBP12)-rapamycin binding domain and has been approved for use as an immunosuppressant by the Food and Drug Administration (FDA). Rapamycin-activated Caspase-9 (RapaCasp9), a synthetic molecule composed of the FKBP12-rapamycin binding (FRB) domain of mTOR, FKBP12, and the catalytic domain of caspase-9, was developed as a safety switch [21]. RapaCasp9 has been shown to act in chimeric antigen receptor (CAR)-modified T cells as a safety switch [21, 22].

Here we developed hypoimmunogenic hiPSCs armed with HLA-G, PD-L1, PD-L2, and RapaCasp9. Our approach has several unique features. Firstly, it can reduce the expression of HLA class Ia molecules without the need for *B2M* gene knockout. Secondly, it can reduce the expression of HLA class II molecules by *RFXANK* gene knockout. Thirdly, it can increase the expression of HLA-G on the cell surface by expressing exogenous HLA-G and B2M. Fourthly, it can mitigate additional rejection by innate and adaptive immune cells by expressing exogenous HLA-G, PD-L1, and PD-L2. Lastly, it includes a safety switch to ensure safety.

## Methods

### Cell line

The hiPSC line was derived from human umbilical cord blood CD34^+^ cells according to published protocols [23] and established in the Lonza Walkersville facility (Lonza Laboratories, Walkersville, MD, USA). The hiPSCs and gene-engineered hiPSCs were maintained in AK03N medium (Ajinomoto Pharma, Tokyo, Japan) on dishes coated with iMatrix511MG (Matrixome, Osaka, Japan) in a 37℃, 5% CO_2_ humidified incubator.

### Generation of HLA class Ia/II DKO hiPSCs

*HLA-A*, *HLA-B*, *HLA-C,* and *RFXANK* genes were inactivated using Cas9 with two-piece guide RNA (gRNA). To prepare two-piece gRNAs, the synthesized crRNA (Thermo Fisher Scientific, Waltham, MA, A35512) was mixed with tracrRNA (Thermo Fisher Scientific, A35512). The sequences of the target region used in this study are listed in Table 1. The two-piece gRNA was mixed with HiFi Cas9 nuclease protein (Integrated DNA Technologies, Coralville, IA) in a ratio of 1:1 to produce a Cas9 ribonucleoprotein (RNP) complex. One hundred picomoles of Cas9 RNP complex were electroporated into 1.0 × 10^6^ hiPSCs in Neon Buffer R using the Neon Transfection System (Thermo Fisher Scientific) with Preset-Program No. 12 (1200 V, 40 ms, 1 pulse). Each Cas9 RNP complex of *HLA-A*, *-B*, and *-C* genes was sequentially electroporated at five-day intervals to obtain HLA class Ia KO clones. After the final electroporation step, to establish clones, the cells were plated in 96-well plates as single cells per well. Twelve days after plating, the cells of each clone were transferred to wells of a 24-well plate and wells of a 96-well plate. The cells in the 96-well plates were used for screening. The cells in the 24-well plates were seeded to 9 cm dishes for further expansion and cryopreserved.

**Table 1:** The sequences of the gRNAs used in this study

Target	Sequence (5’ → 3’)*
*HLA-A* crRNA	ACAGCGACGCCGCGAGCCAGAGG
*HLA-B* crRNA	CCTCCTCCGCGGGTATGACCAGG
*HLA-C* crRNA	AGCGACGCCGCGAGTCCAAGAGG
*RFXANK* crRNA	TGAGACCGTTCGCTTCCTGCTGG

^*^The protospacer adjacent motif (PAM) sequences are underlined

To identify HLA class Ia KO clones, the clones were treated with 25 ng/mL IFN-γ (R&D Systems, Minneapolis, MN) for 3 days and stained with Alexa Fluor 488-labeled anti-HLA class I antibody (clone W6/32, R&D Systems, FAB7098G) and those with drastically decreased expression of HLA class Ia molecules were selected for further screening. Next, the clones were tested for the presence of altered sequences from the cleavage site using the in vitro Cas9 cleavage assay [24]. Briefly, the cells were lysed with Lysis Buffer for PCR (Takara Bio, Kusatsu, Japan) to obtain crude genomic DNA, and the target sites were amplified by PCR using PrimeSTAR GXL Premix (Takara Bio) with the primer sets listed in Table 2. Next, 3 μL of PCR product and 1.6 pmol Cas9 RNP complexes for each gene were mixed and allowed to cleave the PCR product at 37 °C for 60 min. Agarose gel electrophoresis was performed to analyze the length of the PCR product. The clone with uncut PCR product was selected as a homozygous mutant. Finally, HLA class Ia clones with frameshift mutations were selected by analyzing the DNA sequences of the target sites. To confirm the expression by flow cytometry, HLA class Ia KO clones were treated with 25 ng/mL IFN-γ for 3 days and stained with APC-labeled anti-HLA-A/B/C antibody (clone G46-2.6, BD Pharmingen, 562,006).

**Table 2:** The sequences of the primer sets used in this study

Target	Sequence (5’ → 3’)	Product size (bp)
*HLA-A*	Fwd: AATCAGTGTCGTCGCGGTCG	497
	Rev: AGTCTGTGAGTGGGCCTTCAC	
*HLA-B*	Fwd: GAGACACAGATCTCCAAGACCAACA	889
	Rev: CCTGAGAGGAAAAGTCACGGTTC	
*HLA-C*	Fwd: AGGGAAACGGCCTCTGCGGA	329
	Rev: TCTGTGCCTGGCGCTTGTAC	
*RFXANK*	Fwd: ATACCCACTCATGACGTGACCTG	410
	Rev: CAGCCGCATCTCAAAGACAAG	

To obtain HLA class Ia/II DKO clones, HLA class Ia KO clone was electroporated with Cas9 RNP targeting the *RFXANK* gene under the same electroporation conditions described above. The electroporated cells were plated in 96-well plates as single cells per well to establish clones. Twelve days after plating, the cells of each clone were transferred to wells of 24-well plates and 96-well plates. The cells in the 96-well plates were used for screening. The cells in the 24-well plates were seeded to 9 cm dishes for further expansion and cryopreserved. The clones were tested for the presence of altered sequences from the cleavage site using the in vitro Cas9 cleavage assay [24]. The target sites were amplified by PCR with the primer sets listed in Table 2. The clones with uncleaved PCR products only were selected as a homozygous mutant. Finally, *RFXANK* KO clones with frameshift mutations were selected by analyzing the DNA sequences of the target sites.

### Plasmid construction

The piggyBac donor vector (pPB) was created by introducing piggyBac internal tandem repeats (ITRs) and the desired restriction sites into pHSG298 cloning vector (Takara Bio). To introduce gene expression cassettes between the piggyBac ITRs of pPB, the respective coding regions of human *B2M* (NM_004048.2), *HLA-G* (NM_002127.4), *RapaCasp9* [21], and *PDL1-P2A-PDL2* (NM_014143.3 and NM_025239.3) under the control of human *EF1α* promoter, the internal ribosome entry site (IRES) (NC_001479.1: 260..845)—puromycin resistance gene (human codon optimized sequence from pPUR plasmid; U07648.1: 432..1031) cassette flanked by *loxP* sequence (ATAACTTCGTATAGCATACATTATACGAAGTTAT), and human *GH1* poly(A) (NC_000017.11: 63,917,308..63,916,826) were added. To create the PBase expression vector, human codon-optimized *Trichoplusia ni* PBase (J04364.2) (hPBase) under the control of the human *EF1α* promoter, IRES-hygromycin resistance gene (pTK-Hyg plasmid; U40398.1: 2577..1540), and human *GH1* poly(A), were assembled into pHSG298 cloning vector. The fragment of the human *EF1α* promoter was amplified from pBApo-EF1α Pur plasmid (Takara Bio) by PCR. The other fragments were chemically synthesized by Thermo Fisher Scientific.

### Generation of HyPSCs

To obtain HyPSCs, equimolar amounts of four pPB vectors (total 5 μg), and 5 μg hPBase expression vectors were electroporated into 1.0 × 10^6^ HLA class Ia/II DKO cells under the same electroporation conditions described above. The electroporated cells were cultured in a complete AK03N medium (Ajinomoto) supplemented with puromycin and hygromycin (Thermo Fisher Scientific) for one day and then with puromycin for another 6 days to select the cells expressing all transgenes. The selected cells were dissociated into single cells and seeded into 96-well plates at single cell per well density and cultured for approximately 2 weeks. The candidate clones were expanded and finally cryopreserved. Protein expression of transgenes in gene-modified hiPSC clones was confirmed by flow cytometry for detection of the expression of B2M, HLA-G, PD-L1, and PD-L2 molecules. For the detection of the expression of B2M, HLA-G, PD-L1, and PD-L2 molecules, the cells were stained with APC-labeled anti-PD-L1 antibody (1:20; clone MIH1; Invitrogen, 17–5983-42), FITC-labeled anti-PD-L2 antibody (1:10; clone MIH18; Miltenyi Biotec, 130–098-528), PECy7-labeled anti-B2M antibody (1:20; clone 2M2; BioLegend, 316,318), and PE-labeled anti-HLAG antibody (1:100; clone MEM-G/9; Abcam, Cambridge, England, ab24384). The expression of RapaCasp9 in HyPSCs was assessed by western blotting using a Jess Automated Western Blot System (ProteinSimple, San Jose, CA). Cell lysates were extracted using phosphate buffered saline (PBS) supplemented with 0.5% SDS solution (NIPPON GENE, Toyama, Japan) and protease inhibitor cocktail (FUJIFILM Wako Pure Chemicals, Osaka, Japan). The cell lysates were loaded on the Jess/Wes Separation 12–230 kDa 8 × 25 Capillary Cartridges (ProteinSimple). Anti-FKBP12 antibody (Abcam, ab2918) and anti-β-actin antibody (Abcam, ab179467) were used for labeling specific proteins. The bound antibodies were visualized with the anti-rabbit Detection Module without dilution (ProteinSimple) and quantified by Compass for SW software (ProteinSimple). A complete image of the 25 capillaries is available in Fig. S4. The karyotype analysis was done by Sumika Chemical Analysis Service, Ltd. Tokyo, Japan.

### Immunocytochemistry

The HyPSCs were seeded in a 24-well plate at a density of 4.5 × 10^3^ cells per well and cultured for 6 days to allow colonies to form. For fixation, the cells were washed with PBS, then fixed in 4% paraformaldehyde (PFA) phosphate buffer solution (FUJIFILM Wako Pure Chemicals) for 15 min at room temperature, and washed two additional times in PBS. The permeabilization step involved treating the cells with a 0.1% Triton X-100 solution (BioVision Research, Mountain View, CA) in PBS for 20 min at room temperature. The cells were then blocked with Blocking One solution (Nacalai Tesque, Kyoto, Japan) for 60 min at room temperature and stained with Anti-NANOG Rabbit pAb (1:100; ReproCell, Japan, RCAB004P-F) and Anti-OCT-3/4 mouse mAb (1:200; clone C10, Santa Cruz Biotechnology, Santa Cruz, CA, SC-5279), which were diluted in Dako Real Antibody diluent (Agilent Technologies, Santa Clara, CA). Then, the cells were thoroughly washed three times with the Triton X-100 solution (BioVision) and reacted with Alexa Fluor 488 conjugated Donkey anti-Mouse IgG (1:500; Thermo Fisher Scientific, A21202), or Alexa Fluor 546 conjugated Donkey anti-Rabbit IgG (1:500; Thermo Fisher Scientific, A10040), depending on the host species of the primary antibody. The secondary antibodies were prepared in a solution containing 5 μg/mL 4’,6’-diamidino-2-phenylindole (DAPI; Thermo Fisher Scientific) for nuclear staining. The cells underwent a similar washing process as above. Finally, an inverted fluorescence microscope ECLIPSE Ti (Nikon, Japan) equipped with 10 × and 20 × objectives and filters for DAPI, fluorescein isothiocyanate, and Cyanine 3 was used to visualize the cellular staining patterns.

### Alkaline phosphatase staining

Alkaline phosphatase staining was done using a Vector Blue Alkaline Phosphatase Substrate Kit following the manufacturer’s protocol (Vector Laboratories, Burlingame, CA). In brief, 4.5 × 10^3^ HyPSCs were seeded in a 24-well plate and cultured for 6 days. The cells were fixed with 4% PFA (FUJIFILM Wako Pure Chemicals) and stained with a Vector Blue Alkaline Phosphatase Substrate Kit (Vector Laboratories) according to the manufacturer’s instructions. After the staining reaction was stopped, the stained cells were observed under a bright field microscope (Olympus, Tokyo, Japan).

### Cell viability assay

HyPSCs and wild type (WT) hiPSC cells were seeded in a 96-well plate at a density of 5.0 × 10^3^ cells per well. The following day, the culture medium was replaced with a medium containing different concentrations (0.003 nM, 0.01 nM, 0.03 nM, 0.1 nM, 0.3 nM, or 1 nM) of rapamycin (Selleckchem, Munich, Germany). Cell viability was measured at 24 h post-rapamycin treatment using the CellTiter-Glo Luminescent Cell Viability Assay (Promega, Madison, WI), according to the manufacturer’s instructions. Luminescence in each well was measured with a SYNERGY H1 microplate reader (Agilent Technologies). Cell viability was calculated using the luminescence values obtained under the various rapamycin treatment conditions. The viability of untreated WT cells and HyPSCs was used as the reference value (set as 1), and the relative cell viability was calculated by dividing the luminescence value of rapamycin-treated cells by the reference value.

### Tri-lineage differentiation

To generate embryoid bodies, HyPSCs (1.0 × 10^4^ per well) were seeded in a PrimeSurface 96-well U-bottom plate (S-BIO, Tokyo, Japan) and cultured first in StemFit AK03N medium without Liquid C (Ajinomoto) supplemented with 10 μM Y-27632 for a day, and then in StemFit AK03N medium without Liquid C (Ajinomoto) for another 6 days. The obtained embryoid bodies were seeded at a density of 4 embryoid bodies per well in a gelatin-coated 24-well plate and cultured in DMEM (Nakalai Tesque, Kyoto, Japan) supplemented with 10% fetal bovine serum (FBS; CORNING, Corning, NY), 1 × MEM NEAA Solution (Thermo Fisher Scientific) and 1 × GlutaMAX (Thermo Fisher Scientific) for 21 days. The attached cells were washed with PBS, fixed in 4% PFA solution (Nakalai Tesque), permeabilized in 0.2% Triton X-100 (Merck, Darmstadt, Germany), and stained with anti-βIII tubulin antibody (1:500; rabbit polyclonal, Abcam, ab18207), anti-α-SMA antibody (1:10; clone 1A4, Agilent Technologies, IR611), and anti-SOX17 antibody (1:100; goat polyclonal, R&D Systems, AF1924), respectively. The stained cells were washed with PBS and incubated with the secondary antibodies anti-rabbit IgG antibody Alexa 488 (1:1000; Thermo Fisher Sciences, A21206), anti-mouse IgG antibody Alexa 488 (1:1000; Thermo Fisher Sciences, A21202), and anti-goat IgG antibody Alexa 488 (1:1000; Thermo Fisher Sciences, A11055), respectively. Hoechst33342 (Thermo Fisher Sciences) was used for nuclear staining. The fluorescent images of stained cells were captured with a BZ-X710 all-in-one fluorescence microscope (KEYENCE, Osaka, Japan).

### Differentiation to HPCs

Induction of differentiation from hiPSCs into HPCs was performed according to the literature [25]. WT and gene-edited hiPSCs were seeded at a density of 3.0 × 10^3^ cells per dish onto 60 mm dishes coated with iMatrix-511 (Matrixome) and cultured in AK03N (Ajinomoto) supplemented with 10 µM Y-27632 (FUJIFILM Wako Pure Chemical). The following day, the medium was replaced with AK03N (Ajinomoto) and cultured for 6 days. For mesoderm induction, the cells were replaced and cultured in Essential 8 (Thermo Fisher Scientific) supplemented with 80 ng/mL bone morphogenetic protein 4 (BMP4; FUJIFILM Wako Pure Chemical), 2 μM CHIR99021 (FUJIFILM Wako Pure Chemical), and 80 ng/mL vascular endothelial growth factor (VEGF165; FUJIFILM Wako Pure Chemical) for 2 days and in Essential 6 (Thermo Fisher Scientific) supplemented with 80 ng/mL VEGF165 (FUJIFILM Wako Pure Chemical), 50 ng/mL stem cell factor (SCF; FUJIFILM Wako Pure Chemical), and 2 μM SB431542 (FUJIFILM Wako Pure Chemical) for an additional 2 days. To induce HPC differentiation, the cells were replaced and cultured with StemPro-34 SFM (Thermo Fisher Scientific) supplemented with 50 ng/mL SCF (FUJIFILM Wako Pure Chemical) and 50 ng/mL FLT3L (PeproTech, Rocky Hill, NJ) for 10 days. Then, the floating cells that appeared were sequentially collected for another 8 days and the collected cells were cryopreserved. The expression of HPC-specific markers was measured with a MACSQuant Analyzer 10 Flow Cytometer (Miltenyi Biotec, Bergisch Gladbach, Germany) using anti-CD45 antibody (1:20; clone HI30; BD Pharmingen, 563,880) and anti-CD43 antibody (1:20; clone 1G10; BD Pharmingen, 555,475).

### Differentiation to endothelial cells

The method for differentiation of HyPSCs into ECs was based on the literature [26, 27] with modifications. Briefly, 2.5 × 10^6^ HyPSCs were seeded in iMatrix-511 (Matrixome) coated culture dishes containing StemFit AK03N (Ajinomoto) supplemented with 10 µM Y-27632 (FUJIFILM Wako Pure Chemical). The following day, the medium was replaced with a mesoderm induction medium composed of DMEM/F12 (Thermo Fisher Scientific) with 2% B27 Supplement (Thermo Fisher Scientific), 1% GlutaMAX (Thermo Fisher Scientific), 25 ng/mL BMP4 (PeproTech), and 8 µM CHIR99021 (FUJIFILM Wako Pure Chemicals), and the cells were incubated for three days. The mesoderm induction medium was replaced with an EC induction medium composed of StemPro-34 SFM (Thermo Fisher Scientific) supplemented with 2 mM L-Glutamine (Thermo Fisher Scientific), 200 ng/mL VEGF-A (PeproTech) and 2 µM forskolin (Sigma Aldrich), and the cells were incubated for an additional three days. On Day 7 of differentiation, the medium was replaced with an EC induction medium without 2 µM forskolin (Sigma Aldrich). On Day 8 of differentiation, 1.0 × 10^6^ ECs were replated on a fibronectin-coated dish and allowed to proliferate for 2 passages in an EC maintenance medium composed of StemPro-34 SFM (Thermo Fisher Scientific) supplemented with 2 mM L-glutamine (Thermo Fisher Scientific), 50 ng/mL VEGF-A (PeproTech), 20 ng/mL bFGF (PeproTech) and 10 ng/mL epidermal growth factor (EGF; PeproTech). During these steps toward differentiation, the expression levels of several endothelial cell surface molecules that are known markers for endothelial differentiation were monitored by flow cytometry. The following antibodies were used: anti-CD31 antibody (1:5; clone WM59; BD Biosciences, 555,445), anti-CD144 antibody (1:5; clone 55-7H1; BD Biosciences, 560,410), anti-CD34 antibody (1:20; clone 8G12; BD Biosciences, 340,441), anti-CD304 antibody (1:50; clone AD5-17F6; Miltenyi Biotec 130–114-041), anti-CD157 antibody (1:20; clone SY/11B5; BD Biosciences, 564,214), anti-CD140b antibody (1:5; clone 28D4; BD Biosciences, 558,821), and anti-CD30 antibody (1:20; clone BerH8; BD Biosciences, 563,500). We performed functional assays to verify the endothelial nature of the HyPSC-derived ECs by testing for EC capillary tube formation, which is characteristic of the angiogenesis of ECs. The ECs at passage 2 were seeded at a density of 1.0 × 10^5^ cells per well onto a 24-well plate coated with Matrigel (Corning, 354,234) and cultured for 24 h at 37 °C allowing them to form capillary tubes. The formation of capillary-like networks was monitored under a microscope.

### Differentiation to hepatocytes

Hepatic differentiation from HyPSCs was performed according to the published protocols with some modifications [27–29]. Briefly, 6.0 × 10^5^ HyPSCs were seeded onto an iMatrix-511 (Matrixome)-coated 6-well plate and cultured in StemFit AK03N (Ajinomoto) supplemented with 10 µM Y-27632 (FUJIFILM Wako Pure Chemicals). On day 1, the medium was changed to RPMI-1640 (Thermo Fisher Scientific) supplemented with 20% StemFit for Differentiation (Ajinomoto), 3 µM CHIR99021 (FUJIFILM Wako Pure Chemicals), and 100 ng/mL Activin A (PeproTech). Sodium butyrate (0.5 mM; FUJIFILM Wako Pure Chemical) was added from Day 2 to Day 5. On Day 7, the medium was changed to Hepatocyte Culture Medium (HCM) without EGF (Lonza, Bazel, Switzerland) supplemented with 5% FBS (SAFC Biosciences, Melbourne, Australia), 20 ng/mL hepatocyte growth factor (HGF; PeproTech), 20 ng/mL Oncostatin M (PeproTech) and 100 nM dexamethasone (Sigma Aldrich). Albumin-positive cell ratio was evaluated by flow cytometric analysis at the end of culture (Day 30). Anti-albumin antibody (1:1000; clone 188,835; R&D Systems, Minneapolis, MN, IC1455A) was used. The urea synthesis activity, one of the hepatocyte-specific functions, was also assessed to confirm the hepatic differentiation of HyPSCs. Prior to the urea assay, the culture medium was changed to a fresh evaluation medium containing 1 mM ammonium chloride (FUJIFILM Wako Pure Chemical). After 24 h, the evaluation medium was collected, and the cells were harvested from each well with 0.05% Trypsin–EDTA. The urea concentration in the evaluation medium was measured by a QuantiChrom Urea Assay Kit (BioAssay Systems, San Francisco, CA). The urea production of HyPSC-derived hepatocytes was normalized to 1.0 × 10^6^ cells per day.

### T cell proliferation assay

THP-1 cells were treated with 50 ng/mL IFN-γ (R&D Systems) in RPMI1640 (Thermo Fisher Scientific) supplemented with 10% FBS (Biosera, Cholet, France) and 0.05 mM 2-mercaptoethanol (2-ME) for 2 days at a concentration of 4.0 × 10^5^ cells/mL in a T75 flask. Similarly, hiPSC-derived HPCs were treated with 50 ng/mL IFN-γ (R&D Systems) in X-VIVO 15 medium (Lonza) supplemented with 10% Human Serum (Sigma Aldrich), 10 mM HEPES (Thermo Fisher Scientific), 1 × GlutaMAX Supplement (Thermo Fisher Scientific), 1 mM sodium pyruvate (Sigma Aldrich), 1 × MEM NEAA Solution (Thermo Fisher Scientific), 50 ng/mL FLT3L (R&D SYSTEMS), 50 ng/mL SCF (R&D SYSTEMS), and 50 ng/mL TPO (PeproTech) for 2 days at a concentration of 1.0 × 10^6^ cells/mL in a 6-well plate. Then, the THP-1 cells and hiPSC-derived HPCs were treated with 10 µg/mL mitomycin C (Sigma Aldrich) for 2 h and co-cultured with HLA-mismatch human CD3^+^ T cells (Cellero, Lowell, MA, 1017, HLA types are listed in Table 3) in a 48-well plate for 7 days at an effector to target (E/T) ratio of 10:1 (effector: 2.0 × 10^5^ cells, target: 2.0 × 10^4^ cells) in a T cell stimulation medium (RPMI1640 [Thermo Fisher Scientific] supplemented with 10% FBS [Thermo Fisher Scientific], 10 mM HEPES [Thermo Fisher Scientific], 1 mM sodium pyruvate [Thermo Fisher Scientific], 1 × MEM NEAA Solution [Thermo Fisher Scientific], 0.1 mM 2-ME [Sigma Aldrich], 20 U/mL IL-2 [PeproTech], and anti-CD28/CD49d antibody [clone L293; BD Biosciences, 347690]). Then, the cells were labeled with 10 μM 5-ethynyl-2’-deoxyuridine (EdU; Thermo Fisher Scientific) for 2 h, harvested, and stained with anti-CD3 antibody (1:50; clone REA613; Miltenyi Biotec, 130–113-138), anti-CD4 antibody (1:50; clone REA623; Miltenyi Biotec, 130–113-225), and anti-CD8 antibody (1:50; clone BW135/80; Miltenyi Biotec, 130–113-160). After fixation and permeabilization, the cells were EdU labeled with the Click-iT Plus EdU Alexa Fluor 647 Flow Cytometry Assay Kit (Thermo Fisher Scientific) according to the manufacturer’s instructions and the labeled cells were detected by flow cytometry. The percentage of proliferative CD3^+^/CD4^+^ T cells and CD3^+^/CD8^+^ T cells were measured with a MACSQuant Analyzer 10 Flow Cytometer (Miltenyi Biotec).

**Table 3:** HLA types of CD3^+^ T cells and hiPSCs

Sample	HLA	Allele 1	Allele 2
CD3^+^ T cells	HLA-A	01	03
	HLA-B	27	37
	HLA-C	02	06
	HLA-DRB1	01	11
hiPSCs	HLA-A	02	32
	HLA-B	44	44
	HLA-C	05	05
	HLA-DRB1	04	13

### Cytotoxicity by HLA-reactive CD8^+^ T cells

The cytotoxicity assay using HLA-reactive CD8^+^ T cells was performed according to published literature [10]. CD8^+^ T cells were obtained by sorting from CD3^+^ T cells (Cellero) using the EasySep Human CD8^+^ T Cell Isolation Kit (STEMCELL Technologies, Vancouver, BC). The IFN-γ treated WT hiPSC-derived HPCs were treated with 10 µg/mL mitomycin C (Sigma Aldrich) for 2 h. To prime CD8^+^ T cells to allogeneic HLA reactive CD8^+^ T cells, 5.0 × 10^4^ mitomycin-treated HPCs and 5.0 × 10^5^ CD8^+^ T cells were co-cultured in T cell stimulation medium for 7 days in a 96-well round bottom plate. Further, 8.5 × 10^5^ HLA-reactive CD8^+^ T cells were co-cultured with 8.5 × 10^5^ mitomycin-treated HPCs in RPMI1640 (Thermo Fisher Scientific) supplemented with 10% FBS (SAFC Biosciences), 2 µg/mL phytohemagglutinin (Sigma Aldrich), 50 ng/mL IL-7 (PeproTech) and 50 ng/mL IL-15 (PeproTech) for 4 days. To expand the pool of HLA-reactive CD8^+^ T cells, the cells were cultured in RPMI1640 (Thermo Fisher Scientific) supplemented with 10% FBS (SAFC Biosciences), 1 × Insulin-Transferrin-Selenium (Thermo Fisher Scientific), 50 µg/mL L-ascorbic acid (FUJIFILM Wako Pure Chemical), 50 ng/mL IL-7 (PeproTech), and 50 ng/mL IL-15 (PeproTech) for an additional 12 days, while the medium was exchanged every two days to adjust the cell concentration to 1.0 × 10^6^ cells/mL. The IFN-γ treated hiPSC-derived HPCs were stained with 5 μM CellTrace reagent in CellTrace Violet Cell Proliferation Kit (Thermo Fisher Scientific) at a concentration of 2.0 × 10^6^ cells/mL according to the manufacturer’s instructions. Fifty thousand labeled hiPSC-derived HPCs were co-cultured with the HLA-reactive CD8^+^ T cells at E/T ratios of 1:1, 2.5:1, 5:1, 10:1, and 20:1 for 3 h in RPMI1640 (Thermo Fisher Scientific) containing 10% FBS (Thermo Fisher Scientific). The cells were stained with SYTOX Green Dead Cell Stain (Thermo Fisher Scientific) and analyzed with a MACSQuant Analyzer 10 Flow Cytometer (Miltenyi Biotec). To assess the spontaneous death of the target and effector cells, the target cells alone (target cell control) were stained with CellTrace Violet (Invitrogen) and SYTOX Green (Thermo Fisher Scientific), and the effector cells alone (effector cell control) were stained with SYTOX Green (Thermo Fisher Scientific). The analysis was performed by splitting the FITC-A/VioBlue-A (Y-axis/X-axis) dot plot into quadrants based on the target and effecter cell control, followed by counting the number of events in each of four regions (upper right: UR, upper left: UL, lower right: LR, and lower left: LL) of the quadrant gate. The ratio of spontaneous death in the target cell control (DT) was determined by dividing UR by LR of the target cell control, and the ratio of spontaneous death in the effector cell control (DE) was determined by dividing UR by LL of the effector cell control. Cytotoxicity (% of lysis) was calculated by using the following equation.

$${\text{Cytotoxicity }}\left( {{\text{\% of lysis}}} \right) = \frac{{{\text{UR }} - { }\left( {{\text{DT }} \times {\text{ LR }} + {\text{ DE }} \times {\text{ LL}}} \right)}}{{{\text{LR }} + {\text{ UR}} - { }\left( {{\text{ DT }} \times {\text{ LR }} + {\text{ DE }} \times {\text{ LL }}} \right)}} \times 100$$

### Cytotoxicity by NK cells

WT hiPSCs, HLA class Ia/II DKO hiPSCs, and HyPSCs were treated with 25 ng/mL IFN-γ (R&D Systems) in AK03N (Ajinomoto) for 3 days. Then, the hiPSCs and K562 cells were labeled with a Calcein-AM Labeling Kit (Biolegend, San Diego, CA) at a concentration of 1.0 × 10^7^ cells/mL according to the manufacturer’s instructions. The labeled cells were co-cultured with peripheral blood mononuclear cell (PBMC)-derived NK cells (Funakoshi, FN105, Tokyo, Japan) for 2 h at an E/T ratio of 5:1 (effector: 2.5 × 10^5^ cells, target: 5.0 × 10^4^ cells) in 500 μL of AK03N (Ajinomoto) containing 10 mM Y-27632 (FUJIFILM Wako Pure Chemical). The dead cells were stained with SYTOX Red Dead Cell Stain (Thermo Fisher Scientific) and analyzed with a MACSQuant Analyzer 10 Flow Cytometer (Miltenyi Biotec). To assess the spontaneous death of the target and effector cells, the target cells alone were stained with Calcein-AM (Thermo Fisher Scientific) and SYTOX Red (Thermo Fisher Scientific), and the effector cells alone were stained with SYTOX Red (Thermo Fisher Scientific). The analysis was performed as described above, except for splitting the APC-A/FITC-A (Y-axis/X-axis) dot plot.

### Flow cytometry-based phagocytosis assay

The flow cytometry-based phagocytosis assay was performed according to the published protocol with slight modifications [30]. THP-1 cells were differentiated into macrophages by 48 h of incubation with 100 ng/mL phorbol 12-myristate 13-acetate (PMA; Adipogen Corp., San Diego, CA) followed by 48 h of incubation in RPMI medium (Thermo Fisher Scientific) [31]. The macrophages were stimulated by an additional 24 h of incubation with 10 ng/mL IFN-γ (R&D Systems) and 50 pg/mL lipopolysaccharide (LPS; Sigma-Aldrich). Prior to co-culture, target cells and the macrophages were stained with CellTrace Violet (Invitrogen) and CellTrace CFSE (Invitrogen) for each. Then, labeled-target cells were mixed with labeled macrophages in 96-well plates at an E/T ratio of a 1:5 (effector cells: 2.5 × 10^4^ cells, target cells: 1.25 × 10^5^ cells), and cultured for 2 h at 37 °C in a 5% CO_2_ incubator. After co-culture, the cells were detached by TrypLE Express enzyme solution (Thermo Fisher Scientific), stained with Cell Viability Solution (BD Biosciences), and analyzed with a MACSQuant analyzer 10 (Miltenyi Biotec). The analysis was performed as described above, except for splitting the VioBlue-A/FITC-A (Y-axis/X-axis) dot plot. The ratio of the background staining in the target cell (NOISE_T_) was determined by dividing UR by UL of the target cell alone, and the ratio of the background staining in the effector cell (NOISE_E_) was determined by dividing UR by LR of the effector cell alone. Phagocytosis (%) was calculated by using the following equation.

$$Phagocytosis\left( \% \right) = \frac{{UR - \left( {LR \times Noise_{E} + UL \times Noise_{T} } \right)}}{{UL + UR - \left( {LR \times Noise_{E} + UL \times Noise_{T} } \right)}} \times 100$$

### Mice

NOD/Shi-scid IL2rgamma(null) (NOG) mice were purchased from In-Vivo Science Inc. (Kawasaki, Japan) and were kept in animal facilities under pathogen-free conditions with ad libitum access to water and food. All animal experiments were performed in accordance with the relevant institutional and national guidelines and regulations.

## In vivo experiments

The experimental procedures were approved by the Institutional Animal Care and Use Committee of the HEALIOS K.K. (IACUC 1034). We performed the animal experiments according to the ARRIVE guidelines for the reporting of animal experiments. Teratoma formation was performed as the published protocol with slight modifications [32]. Seven-week-old female NOG mice (n = 24) were subcutaneously inoculated with hiPSCs (3.0 × 10^6^ cells/50 μL) transfected with or without constructs containing the *RapaCasp9* gene suspended in an equal volume of Matrigel (Corning). The mice were injected intraperitoneally with 0.16 mg/100 μL of rapamycin (Selleckchem) or vehicle alone (10% EtOH/5% PEG400/5% Tween 80) for 21 days from when the size of the hiPSC-derived teratomas reached 15–25 mm^3^. Teratoma size was measured with a digital caliper (Mitutoyo, Osaka, Japan), and teratoma volume was calculated using the following formula: teratoma volume (mm^3^) = (length × width^2^ × 0.5). On the final day, mice were anesthetized by inhalation of vaporized isoflurane and euthanized by exsanguination from the inferior vena cava, and the weight of the teratomas was measured. Teratomas were fixed in a 10% phosphate-buffered formalin solution, embedded in paraffin, sectioned, stained with hematoxylin/eosin, and viewed microscopically.

### Statistical analysis

All data are presented as the mean ± standard deviation (SD). Comparisons involving multiple groups were performed using a one-way analysis of variance (ANOVA) with a Dunnett post hoc test. The Student or Welch *t*-test was used to evaluate statistically significant differences. All graphs were generated with Excel (Microsoft). Statistical significance was determined at a significance level of *p* < 0.05.

## Results

### Generation of HLA class Ia/II double knockout (DKO) hiPSCs

**Fig. 1:**
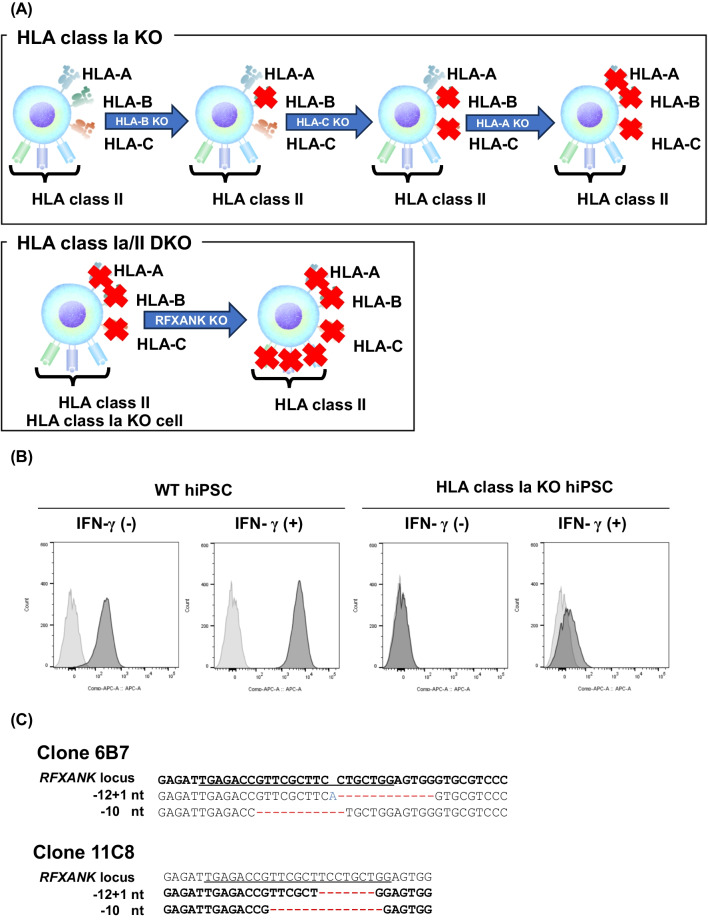
Generation of HLA class Ia/II DKO cells. **A** Procedure of generation of HLA class Ia/II DKO clones. *HLA-A*, *HLA-B*, and *HLA-C* genes were knocked out sequentially to generate HLA class Ia KO cells. *RFXANK* gene was knocked out to generate HLA class Ia/II DKO cells. **B** The expression of HLA class Ia molecules was diminished in HLA class Ia KO cells. The expression of HLA class I molecules in the presence or absence of IFN-γ was tested by flow cytometry using anti-HLA class I antibody (clone G46-2.6). **C** The target DNA sequence of the *RFXANK* gene in HLA class Ia/II DKO clones (6B7 and 11C8). The target region of the *RFXANK* locus was amplified by PCR and used in Sanger sequencing. The deletion is indicated in red, and the insertions are indicated in blue. The sequence of gRNA used is underlined

A major strategy to reduce the surface expression of HLA class I molecules is inactivating *B2M* gene. Since HLA class Ib molecules are key factors in preventing NK cell-mediated lysis, we knocked out the HLA class Ia genes to preserve the cell surface expression of B2M and HLA class Ib molecules. We knocked out the *HLA-A*, *HLA-B*, and *HLA-C* genes sequentially in hiPSCs (Fig. 1A). Firstly, we introduced the *HLA-B*- guide RNA (gRNA)-Cas9 complex into the hiPSCs via electroporation. Five days later, we introduced the *HLA-C*-gRNA-Cas9 complex, and 5 days after that, we introduced the *HLA-A*-gRNA-Cas9 complex. Through this procedure, no clone was found to have mutations at all six target sites, but two clones (clone 18B12 and 17E10) were confirmed to have frameshift mutations at five target sites (Fig. S1A). Therefore, we performed additional editing steps to knock out the residual *HLA-A* locus of clone 18B12. The *HLA-A*-gRNA-Cas9 complex was electroporated into clone 18B12 and a total of 75 clones were isolated. Finally, we obtained an HLA class Ia knockout (KO) clone in which the expression of HLA-A/B/C molecules was diminished and biallelic frameshift mutations were present at *HLA-A/B/C* gene loci (Figs. 1B and S1B).

To diminish the expression of HLA class II molecules, the *RFXANK* gene, a transcription factor that controls the expression of the HLA class II genes, was inactivated (Fig. 1A). In brief, the *RFXANK*-gRNA-Cas9 complex was electroporated into HLA class Ia KO cells, and a total of 256 clones were subjected to the in vitro Cas9 cleavage assay and then Sanger sequencing. Finally, we obtained two clones (6B7 and 11C8) that carried biallelic frameshift mutations at the *RFXANK* locus (Fig. 1C). We chose clone 6B7 for further editing.

### Integration of immunomodulatory factors and suicide gene into HLA class Ia/II DKO cells

**Fig. 2:**
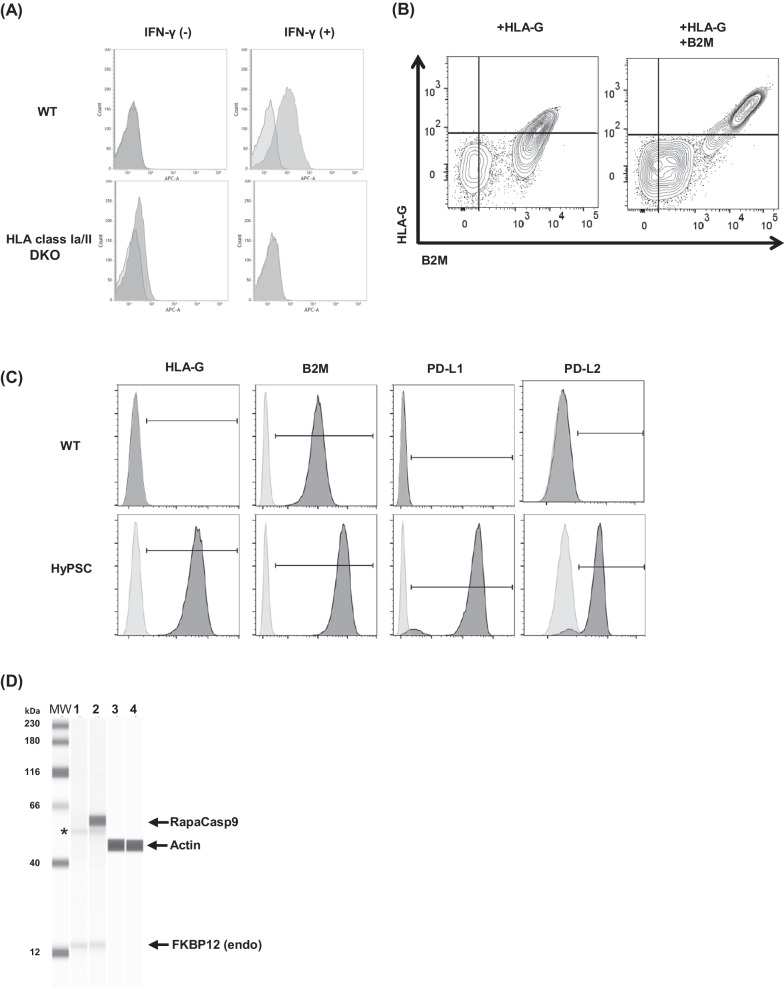
Generation of HyPSCs. **A** No HLA-E expression was observed in HLA class Ia/II DKO cells. The expression of HLA-E in HLA class Ia/II DKO cells was analyzed by flow cytometry. **B** Overexpression of B2M increased HLA-G expression in HLA class Ia/II DKO cells. Flow cytometry was used to analyze the expression of B2M and HLA-G in HLA class Ia/II DKO cells transfected with *HLA-G* gene alone (left) or with *HLA-G* gene and *B2M* gene (right). **C** HyPSCs expressed HLA-G, B2M, PD-L1, and PD-L2 on their cell surface. The expression of transgene was analyzed by flow cytometry in WT hiPSCs and HyPSCs. Light gray histograms represent isotype IgG control and dark gray histograms indicate specific antibody staining. **D** HyPSCs expressed RapaCasp9. The expression of RapaCasp9 was analyzed by a Jess western blot system (ProteinSimple) using anti-FKBP12 antibody in WT hiPSCs (lane 1) and HyPSCs (lane 2). β-Actin was an internal loading control (lane 3 for WT hiPSCs and lane 4 for HyPSCs). The asterisk indicates a nonspecific band resulting from anti-FKBP12 antibody. *MW* molecular weight markers

The cells lacking the expression of HLA class Ia molecules are susceptible to NK cell-mediated lysis by missing self-recognition [13]. Non-classical HLA class Ib molecules, HLA-E and HLA-G, are key inhibitory ligands of NK cell-mediated lysis [33, 34]. We found that the expression of HLA-E was diminished in our HLA class Ia/II DKO clone, even though the *B2M* gene was kept intact (Fig. 2A). The surface expression of HLA-E is controlled by the signal peptide of HLA class Ia [35]. Thus, we thought that the forced expression of HLA-G might give NK cell tolerance to HLA class Ia/II DKO cells instead of HLA-E. HLA-G belongs to non-classical HLA class Ib molecules, and its expression is limited to trophoblast cells and placental chorionic endothelium [36, 37]. It is known that cell surface HLA-G molecules on donor cells result in a more efficient escape from host NK cell-mediated lysis [33]. The forced expression of HLA-G in HLA class Ia/II DKO cells resulted in only a slight increase in surface expression (Fig. 2B left panel). To increase the surface expression of HLA-G, we tested co-transfection of *B2M* gene with the *HLA-G* gene. As expected, additional expression of B2M increased the surface expression of HLA-G on HLA class Ia/II DKO cells (Fig. 2B right panel). Based on these observations, we employed both *HLA-G* and *B2M* genes to mitigate the NK cell-mediated lysis against HLA class Ia/II DKO cells.

To suppress residual T cell activity, we introduced PD-L1 and PD-L2 molecules to directly inhibit T cell response. In addition to this, we introduced RapaCasp9 as a safety switch [21]. RapaCasp9 is a single protein composed of the FRB domain of mTOR, FKBP12, and the catalytic domain of caspase-9 [21]. We introduced four transgene cassettes expressing *HLA-G, B2M, PD-L1-P2A-PD-L2*, and *RapaCasp9* genes under the control of *Elongation factor 1-alpha 1* (*EF1a*) promoter using the piggyBac system [38]. In brief, human codon-optimized piggyBac transposase (hPBase) as well as piggyBac donor DNAs were electroporated into HLA class Ia/II DKO cells. Finally, we obtained an HLA class Ia/II DKO clone expressing HLA-G, B2M, PD-L1, PD-L2, and RapaCasp9 molecules (hereafter designated Hypo-immunogenic pluripotent stem cells, HyPSCs, Figs. 2C and 2D).

### HyPSCs maintain pluripotency and have tri-lineage differentiation potential

**Fig. 3:**
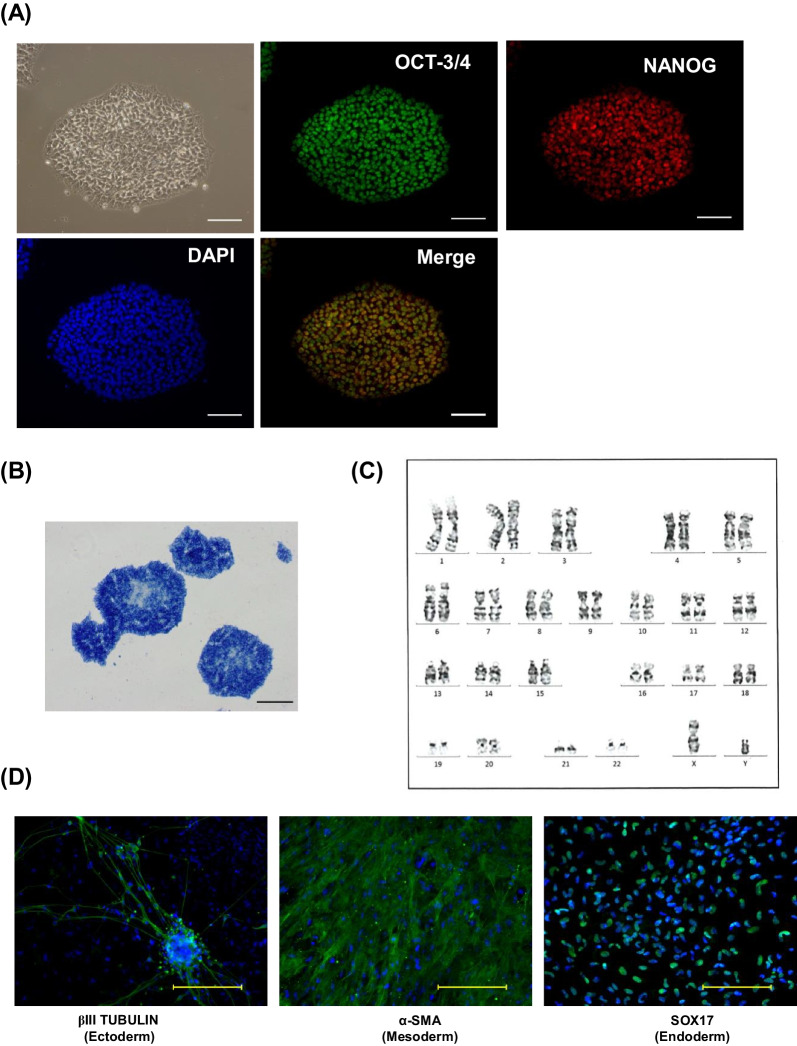
The HyPSC clone retains pluripotency and trilineage differentiation capability. **A** HyPSCs expressed typical pluripotency markers. HyPSCs were immunostained with the pluripotency markers, OCT-3/4 (green) and NANOG (red). The nuclei of HyPSCs were counterstained with DAPI (blue). White scale bar, 100 μm. **B** HyPSCs exhibited alkaline phosphatase activity. Black scale bar, 100 μm. **C** G-band analysis of chromosomes in HyPSCs demonstrated normal karyotype (46, XY). **D** HyPSCs possessed the capacity to differentiate towards ectoderm, mesoderm, and endoderm lineages. The images show HyPSC derivatives stained with antibodies to the trilineage differentiation markers, βIII TUBULIN for ectoderm (left), α-SMA for mesoderm (middle), and SOX17 for endoderm (right). The nuclei of the differentiated cells were counterstained with Hoechst33342 (blue). Yellow scale bar, 100 μm

To ask whether the genetic engineering and single-cell cloning steps affect pluripotency, we assessed the expression of pluripotent stem cell markers and the alkaline phosphatase activity of HyPSCs. We confirmed that HyPSCs maintain the expression of OCT-3/4 and NANOG and alkaline phosphatase activity (Figs. 3A and B). The transgene expression in HyPSCs was maintained for more than 180 days under maintenance culture conditions (Fig. S2). We also confirmed that HyPSCs maintained normal karyotype by G-band analysis (Fig. 3C).

Next, we assessed whether HyPSCs possess the capabilities of trilineage differentiation by embryoid body formation. The embryoid bodies generated from HyPSCs were stained with anti-βIII Tubulin antibody (ectoderm marker), anti-α-smooth muscle actin (SMA) antibody (mesoderm marker), and anti-SOX17 antibody (endoderm marker), respectively (Fig. 3D). The HyPSC-derived differentiated cells were expressing all trilineage differentiation markers. We confirmed that HyPSCs maintain pluripotency as well as tri-lineage differentiation competency.

**Fig. 4:**
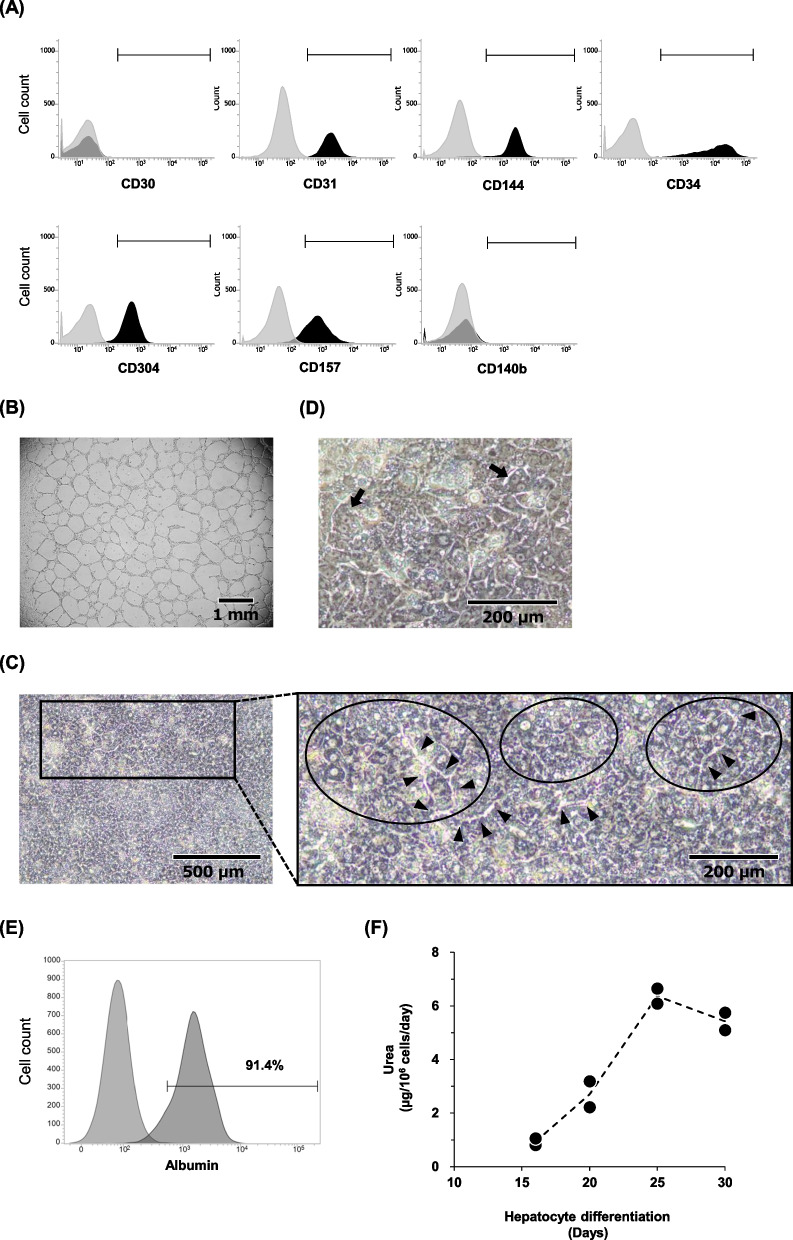
HyPSC clones can differentiate into functional endothelial cells and hepatocytes. **A** HyPSC-derived ECs expressed typical endothelial cell markers. The expression of endothelial cell markers on HyPSC-derived ECs was analyzed by flow cytometry. Gray histograms represent the unstained cells as the isotype IgG control and black histograms represent the cells stained with specific antibodies. **B** HyPSC-derived ECs formed tubular structures. The function of HyPSC-derived ECs was assessed by the 2D tube forming assay. Black scale bar, 1 mm. **C** HyPSCs differentiated into hepatocytes. A representative image of the HyPSC-derived hepatocytes is shown (left). The black circles and arrowheads in the high-magnified image (right) indicate the typical rounded polygonal cobblestone-like morphology and the bile canalicular-like structures, respectively. Black scale bars, 500 µm and 200 µm for the low- and high-magnified images, respectively. **D** HyPSC-derived hepatocytes formed binuclear cells. The black arrows indicate the binuclear cells in the re-plated HyPSC-derived hepatocytes. Black scale bar, 200 µm. **E** HyPSC-derived hepatocytes produced albumin. The expression of albumin in HyPSC-derived hepatocytes was analyzed by flow cytometry. **F** Production of urea in HyPSC-derived cells increased upon hepatic differentiation. The data at days 16, 20, 25, and 30 from two independent experiments were plotted. The dotted line represents the trend line for the mean value (n = 2)

Further, we tested the capability of HyPSCs to differentiate into functional cells. We induced HyPSCs to differentiate into endothelial cells (ECs). The differentiated cells expressed the general EC markers (CD31, CD144, CD34), angiogenic EC marker (CD304), and endothelial stem/progenitor marker (CD157) but not the mural cell marker (CD140b) and undifferentiated cell marker (CD30) (Fig. 4A). These induced ECs could form a tube-like structure on Matrigel, showing the functional properties of ECs (Fig. 4B). Next, we differentiated HyPSCs into hepatocytes and evaluated their hepatic function. We observed rounded polygonal cells growing in cobblestone-like clusters, each cell with a clear nucleus, which is a morphological feature of iPSC-derived hepatocytes [27], as well as differentiated cells forming bile canalicular-like structures (Fig. 4C). Additionally, we observed the presence of binuclear cells in the re-plated HyPSC-derived hepatocytes (Fig. 4D) and the expression of albumin, a hepatocyte marker, in the most differentiated cells (Fig. 4E). Urea synthesis is one of the key functions of hepatocytes and the major pathway for ammonia detoxification in the liver. During hepatic differentiation, we evaluated the urea synthesis ability of the HyPSC-derived cells. We found that the HyPSC-derived cells began producing urea on Day 16, and the urea production increased with the duration of the culture (Fig. 4F). These results demonstrate that HyPSCs can differentiate into functional endothelial cells and hepatocytes. In addition to this, the differentiation of HyPSCs into otic progenitor cells has been reported [39].

### HyPSC-derived cells can evade adaptive and innate immunity

**Fig. 5:**
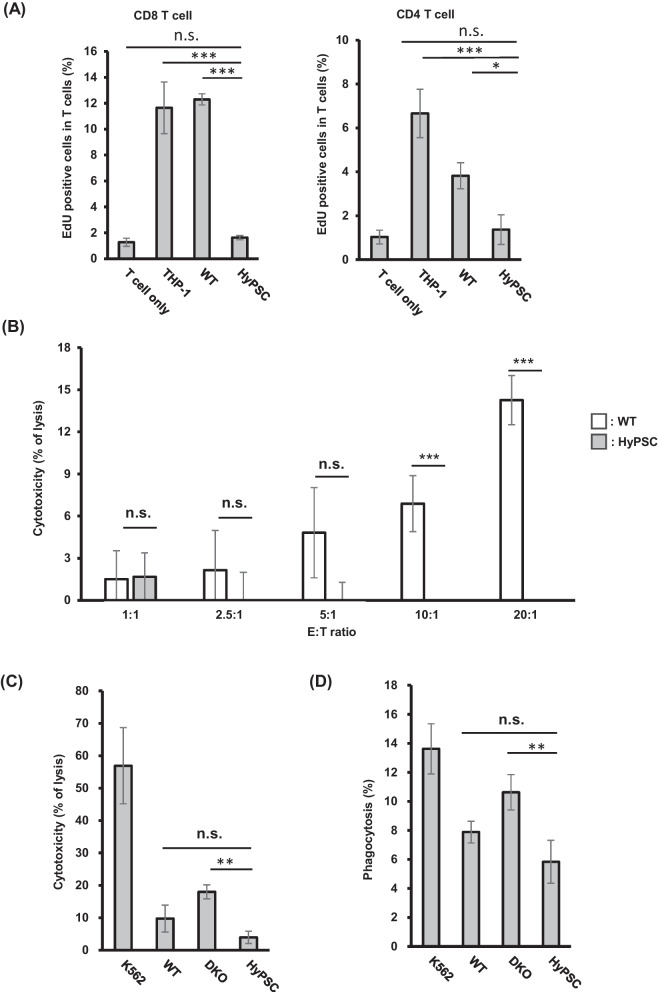
HyPSC-derived cells evaded innate and adaptive immunity. **A** HyPSC-derived HPCs evaded the CD8^+^ T cell- (left) and CD4^+^ T cell- (right) mediated immune responses. Bar plot represents the percentage of activated T cells co-cultured with THP-1 cells, WT hiPSCs, and HyPSC derivatives. The values for THP-1 cells and WT hiPSCs were compared to the value for HyPSC-derived HPCs by a one-way ANOVA with Dunnett post hoc test (* *p* < 0.05, *** *p* < 0.001, n.s. no significance). **B** HyPSC-derived HPCs evaded immunity mediated by HLA-reactive CD8^+^ T cells. Bar plot represents the percentage of CD8^+^ cell-mediated cytotoxicity against WT hiPSCs, and HyPSCs. The difference between WT hiPSC-derived and HyPSC-derived HPCs was evaluated with the Student *t*-test (*** *p* < 0.001, n.s. no significance). **C** HyPSCs exhibited tolerance to NK cell-mediated killing. Bar plot represents the percentage of NK cell-mediated cytotoxicity against WT hiPSCs, HLA class Ia/II DKO cells, and HyPSCs. K562 cells were used as a positive control for HLA class I deficiency. The values for WT hiPSCs and HLA class Ia/II DKO cells were compared to the value for HyPSCs by a one-way ANOVA with Dunnett post hoc test (** *p* < 0.01, n.s. no significance). **D** HyPSC-derived HPCs evaded phagocytosis by THP-1 derived macrophages. Bar plot represents the percentage of phagocytosed WT hiPSCs, HLA class Ia/II DKO cells, and HyPSCs. K562 cells were used as a positive control. The percentages of phagocytosed WT hiPSCs and HLA class Ia/II DKO cells were compared to that of HyPSCs by a one-way ANOVA with Dunnett post hoc test (** *p* < 0.01, n.s. no significance)

To mitigate immune rejection against donor cells by host T cells caused by HLA mismatch, HyPSCs were engineered to have diminished expression of HLA class Ia and HLA class II molecules, as well as forced expression of immune checkpoint proteins PD-L1 and PD-L2 for additional mitigation. We asked whether HyPSCs mitigate the immune rejection of T cells. We performed a mixed lymphoid reaction assay with HLA-mismatched CD3^+^ T cells (Table 3). We used HPCs differentiated from HyPSCs (Fig. S3A) and confirmed the lack of expression of HLA-A, -B, -C, and HLA class II molecules (Fig. S3B), and the expression of transgenes on their surface (Fig. S3C). As expected, HLA-mismatch CD8^+^ and CD4^+^ T cells showed almost no response against HyPSC-derived HPCs, while they showed a statistically significant response against HLA-expressing WT hiPSC-derived HPCs compared to effector T cells alone (Fig. 5A). Next, we asked whether HyPSC-derived HPCs can evade CD8^+^ T cell cytotoxicity. We generated HLA-reactive CD8^+^ T cells that recognize HLA molecules expressed on WT hiPSCs. Then, we assessed the cytotoxicity of the HLA-reactive CD8^+^ T cells against HyPSC-derived HPCs (Fig. 5B). As expected, HyPSC-derived HPCs were less vulnerable to attack by HLA-reactive CD8^+^ T cells, compared to WT hiPSC-derived HPCs which were lysed in dose-dependent manner. These data demonstrate that HyPSC-derived cells evade allogenic T cell immune rejection.

It is known that reducing the expression of HLA class I molecules on the surface of donor cells can help protect them from being attacked by host T cells due to HLA mismatch. However, when there is a lack of HLA class I molecules on the surface of donor cells, they become vulnerable to attack by host NK cells. To test whether HyPSCs can evade NK cell rejection, we assessed the NK cell cytotoxicity against HyPSCs (Fig. 5C). As expected, NK cells lysed more HLA class Ia/II DKO cells compared to WT hiPSCs. While HyPSCs were lysed to a lesser extent compared to WT hiPSCs. These data demonstrate that forced expression of HLA-G enables HyPSCs to escape from host NK cell rejection.

HLA class I molecules on the cell surface directly protect the cells from phagocytosis by macrophages via the inhibitory receptor LILRB1 [30]. LILRB1 can bind to B2M-associated HLA-G [40, 41]. Therefore, we asked whether HyPSCs can mitigate the elimination from macrophages via HLA-G molecules. Phagocytotic macrophages were differentiated from THP-1 by PMA and activated with IFN-γ and LPS. We mixed the activated macrophages with HLA class I/II DKO-derived or HyPSC-derived HPCs and assessed their phagocytic activity. The macrophages showed higher phagocytic activity when they were mixed with HLA class Ia/II DKO-derived HPCs or K562 cells that were not expressing HLA class I molecules. In contrast, HyPSC-derived HPCs were insusceptible to the phagocytic activity of macrophages (Fig. 5D). These data demonstrate that HLA-G expression endows the HyPSCs with the ability to escape from phagocytosis by host macrophages.

### RapaCasp9 can work as a safety switch in HyPSCs and its derivatives

**Fig. 6:**
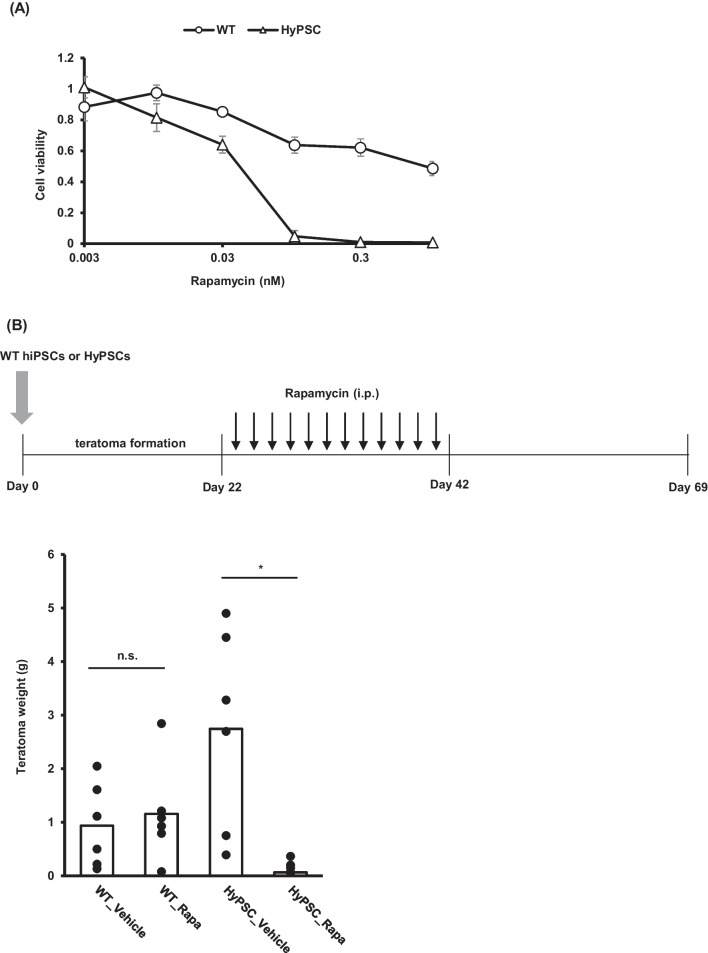
A safety switch works in HyPSC cells, both in vitro and in vivo. **A** Rapamycin induced apoptosis in HyPSCs in dose-dependent manner. WT hiPSCs and HyPSCs were treated with increasing concentrations of rapamycin (0.003 to 0.1 μM). Cell viability was measured by the ATP assay. The data are presented as mean ± SD (n = 3). **B** Administration of rapamycin resulted in the disappearance of hiPSC-derived teratomas, whereas WT hiPSC-derived teratomas persisted. Teratoma weights at day 69 were measured (n = 6). The difference between samples was analyzed using the Welch *t-*test (* *p* < 0.05, n.s. no significance)

As described above, HyPSCs can escape both from innate and adaptive immune systems. One concern is that it would be difficult to eliminate HyPSC-derived cells from the host immune system when they cause issues after transplantation. To mitigate this risk, we introduced RapaCasp9 as a safety switch. RapaCasp9 is a synthetic molecule composed of the FRB domain of mTOR, FKBP12, and the catalytic domain of caspase-9 [21]. RapaCasp9 can be activated by forming a homodimer in the presence of rapamycin, and the homodimer activates the apoptosis signaling pathway through caspase-3/-7. We tested whether rapamycin could induce apoptosis in HyPSCs. Treatment of rapamycin demonstrated that apoptosis was induced in HyPSCs in a dose-dependent manner (Fig. 6A). As rapamycin is an inhibitor of mTOR and affects cell proliferation, rapamycin treatment showed growth inhibition of WT hiPSCs (Fig. 6A). To test rapamycin-induced apoptosis in HyPSCs, we implanted HyPSCs subcutaneously into NOG mice and treated them with rapamycin for 22 days after transplantation. The teratoma of mice transplanted with WT hiPSCs continued to grow even after rapamycin intraperitoneal injection, whereas the teratoma of mice transplanted with HyPSCs was reduced in size (Fig. 6B). This result demonstrated that rapamycin could induce apoptosis in cells differentiated from HyPSCs in vivo. We confirmed that RapaCasp9 served as a safety switch in both in vitro and in vivo settings.

## Discussion

We generated a hypoimmunogenic pluripotent stem cell clone equipped with HLA-G, PD-L1, PD-L2, and RapaCasp9 molecules. This hypoimmunogenic pluripotent stem cell clone has innate and adaptive immune tolerance and differentiates into all three germ layers.

The major strategy for generating hypoimmunogenic cells is disruption of the *B2M* gene, which is responsible for the cell surface expression of HLA class Ia and Ib molecules. With the loss of cell surface expression of HLA class Ia and Ib molecules, cells acquire the ability to evade recognition by CD8^+^ T cells [7–9, 11]. However, HLA class Ia and Ib molecules act as gatekeepers of innate immunity, and NK cells and macrophages eliminate cells that fail to express HLA class I molecules [30, 42]. We demonstrated that cells lacking HLA class Ia molecules can acquire innate immune tolerance by the forced expression of HLA-G. HLA-G can bind to LILRB1, an HLA class I molecule receptor expressed in numerous immune cells including NK cells and macrophages, and acts as a receptor of the “Don’t eat me signal” [43–46]. Therefore, we chose to delete only HLA class Ia genes instead of the *B2M* gene to maintain the HLA class Ib gene expression on HLA class Ia KO cells and force the expression of HLA-G molecules in HyPSCs. Moreover, we found that introducing additional *B2M* genes along with the *HLA-G* gene leads to enhanced cell surface expression of HLA-G molecules. Structural analysis has shown that HLA-G homodimers expose the LILRB1 binding sites of α3 chain, making them accessible to receptors and increasing the avidity for LILRB1 [47, 48]. We speculate that a high expression of the B2M-HLA-G complex could form homodimers on the cell surface, which would prevent elimination by macrophages and NK cells through the LILRB1 signal.

The *RFNANK* gene is one of the genes in the transcriptional complex responsible for activating HLA class II gene expression, and its mutation is frequently found among patients with HLA class II deficiency [49]. Therefore, we chose the *RFXANK* gene to reduce HLA class II expression in HyPSCs and demonstrated that the *RFXANK* gene knockout, similar to *CIITA* gene knockout, another strategy to reduce HLA class II expression, is sufficient to diminish HLA class II expression and evade recognition by CD4^+^ T cells.

It is interesting to note that HyPSCs tended to form larger teratomas than WT hiPSCs in NOG mice (Fig. 6B). The doubling time of WT hiPSCs and HyPSCs is 21.6 ± 2.1 and 24.1 ± 2.4 h, respectively. Thus, the size of teratomas in NOG mice seems not to depend on the proliferation of the transplanted cells. Although NOG mice lack certain immune cells like T, B, and NK cells, they still possess innate immune cells like macrophages and neutrophils, which can trigger antibody-dependent cell cytotoxicity [50]. In addition, depletion of granulocytes can improve the engraftment of human skin in IL-2 NOG mice [51]. This suggests that some xenorejection reactions are still present in NOG mice, and HyPSCs acquired a tolerance to such immune reactions. Since hPD-L1 binding to mPD-1 has been reported [52], we think that this tolerance is accomplished by the binding of hPD-L1 expressed on HyPSCs to PD-1 (mPD-1) present on the surface of mouse granulocytes and macrophages. Human PD-1 (hPD-1) expressed on innate immune cells including NK cells, macrophages, and granulocytes has been reported to contribute to the immune suppressive environment [53–55]. Therefore, we suppose that the combination of hPD-1/hPD-L1 and the HLA-G/ILRB1 axis has significant effects on the allo-tolerance and allo-rejection reactions of innate immune cells.

A major concern of using hypoimmunogenic stem cells in therapy is their safety. The engrafted cells derived from these stem cells can evade the host immune surveillance, increasing the risk of tumorigenicity. To mitigate this risk, safety switches have been utilized as a safety switch [6, 17, 56]. Inducible caspase-9 (iCaspase9) is a synthetic molecule that induces apoptosis by forcing the dimerization of the catalytic domain of caspase-9 with CIDs such as the FK506 dimerizer (AP1903) or rapamycin and has an advantage over the well-studied suicide gene *HSV-TK* in its immunogenicity and rapid activation [22, 57]. In addition, rapamycin is an FDA-approved drug known to inhibit the proliferation of tumor cells by blocking the mTOR signal [58]. Therefore, we integrated the RapaCasp9 system into our HyPSCs to avoid the tumorigenicity risk after transplantation. We demonstrated that the RapaCasp9 system works as a safety switch in HyPSCs and their derivatives in vitro and in vivo. RapaCasp9 system will provide an extra layer of safety for using HyPSCs clinically.

Taniguchi and colleagues demonstrated that organoids composed of hiPSC-derived hepatocytes, endothelial cells, and mesenchymal stem cells are functional and have long persistence in vivo [26, 27]. Their results suggest that organoids composed of hiPSC-derived cells are alternative sources of organ transplantation and that the vascularization of endothelial cells is important for the persistence of organoids. However, the usage of allogenic hiPSCs is still challenging because of their immunogenicity since the vascular endothelial cell is known to express a high level of HLA class I and class II molecules on its surface and is a primary target of allograft rejection in organ transplantation [59, 60]. Interestingly, HyPSCs and other hypoimmunogenic stem cells are demonstrated to have the potential to differentiate into tri-lineage cells including vascular endothelial cells (in this paper, [8, 10]). We believe that hypoimmunogenic stem cells will become an attractive cell source of organoids for regenerative medicine.

## Conclusions

As described above, HyPSCs were less vulnerable to immune rejection by host immune cells and able to differentiate into three germ layer lineages. Moreover, rapamycin administration was able to induce apoptosis of HyPSC-derived cells both in vitro and in vivo. We believe that hypoimmunogenic hiPSCs armed with HLA-G, B2M, PD-L1, PD-L2, and RapaCasp9 molecules might be a good candidate starting material for regenerative therapy.

### Supplementary Information

Additional file 1.

## Data Availability

All data supporting the findings of this study are available within the paper and its Supplementary Information. The authors will provide materials upon request under material transfer agreements (MTAs).

## Abbreviations

ANOVA: Analysis of variance
B2M: β-2-microglobulin
BMP4: Bone morphogenetic protein 4
CID: Chemical inducer of dimerization
CRISPR: Clustered regularly interspaced short palindromic repeats
crRNA: CRISPR RNA
DAPI: 4’,6’-diamidino-2-phenylindole
DE: Definitive endoderm
DKO: Double knockout
EF1α: Elongation factor 1 alpha
EC: Endothelial cell
FBS: Fetal bovine serum
FKBP12: FK506 binding protein 12
FRB: FKBP12-rapamycin binding
FDA: Food and Drug Administration
gRNA: Guide RNA
HSV-TK: Herpes simplex virus thymidine kinase
HPC: Hematopoietic progenitor cell
HCM: Hepatocyte culture medium
HGF: Hepatocyte growth factor
HLA: Human leukocyte antigen
HyPSC: Hypoimmunogenic pluripotent stem cell
iPSC: Induced pluripotent stem cell
IRES: Internal ribosome entry site
KO: Knock out
LPS: Lipopolysaccharide
mTOR: Mechanistic target of rapamycin
NK: Natural killer
NOG: NOD/Shi-scid IL2rgamma(null)
NEAA: Non-essential amino acids
PFA: Paraformaldehyde
PBMC: Peripheral blood mononuclear cell
pPB: PiggyBac donor vector
PBase: PiggyBac transposase
PMA: Phorbol 12-myristate 13-acetate
PBS: Phosphate buffered saline
RapaCasp9: Rapamycin-activated Caspase 9
RNP: Ribonucleoprotein
SD: Standard deviation
SCF: Stem cell factor
WT: Wild type
