# Supplementary material for: Hypoimmunogenic human iPSCs expressing HLA-G, PD-L1, and PD-L2 evade innate and adaptive immunity
Source: Stem Cell Res Ther. 2024 Jul 2;15:193. doi: 10.1186/s13287-024-03810-4 (PMC11218117; doi:10.1186/s13287-024-03810-4)
Supplement: Supplementary file 1 — Additional file 1. [file 13287_2024_3810_MOESM1_ESM.pdf]

(A)

**Clone 18B12**

*HLA-A* **locus** **GTTYGACAGCGACGCCGCGAGCC AGAGGATGGAGCCGCGGGCGCCGT**
**WT** GTTTGACAGCGACGCCGCGAGCC AGAGGATGGAGCCGCGGGCGCCGT
**+1 nt** GTTCGACAGCGACGCCGCGAGCCCAGAGGATGGAGCCGCGGGCGCCGT

*HLA-B* **locus** **GGGCGCCTCCTCCGCGGGTATG ACCAGGACGCCTACGACGGCAAGGATTACA**
**-10 nt** GGGCGCCTCCTC---------- ACCAGGACGCCTACGACGGCAAGGATTACA
**-1+17 nt** GGGCGCCTCCTCCGCGGGTAT-ACCTCCTCCGCGGGTATACCAGGACGCCTA

*HLA-C* **locus** **TCGACAGCGACGCCGCGA GTCCAAGAGGGGAGCCGCGGGCGCCGTGGGTGGAGCA**
**+1 nt** TCGACAGCGACGCCGCGAAcTgCAcGAGGGGAGtCGCGGGCGCCGTGGGTGGAGCA
**-13 nt** TCGACAGCGACGCCGC-- -----------GAGCCGCGGGCGCCGTGGGTGGAGCA

**Clone 17E10**

*HLA-A* **locus** **GTTYGACAGCGACGCCGCGAGCC AGAGGATGGAGCCGCGGGCGCCGT**
**+1 nt** GTTYGACAGCGACGCCGCGAGCCCAGAGGATGGAGCCGCGGGCGCCGT
**-13 nt** GTTYGACAGCGACGCCGCGAGCC -------------GCGGGCGCCGT

*HLA-B* **locus** **GGGCGCCTCCTCCGCGGGTAT GACCAGGACGCCTACGACGGCAAGGATTACA**
**wt** GGGCGCCTCCTCCGCGGGTAT GACCAGGACGCCTACGACGGCAAGGATTACA
**+2 nt** GGGCGCCTCCTCCGCGGGTATATGACCAGGACGCCTACGACGGCAAGGATTACA

*HLA-C* **locus** **TCGACAGCGACGCCGCGAGTCCAAGAGGGGAGCCGCGGGCGCCGTGGGTGGAGCA**
**+1 nt** TCGACAGCGACGCCGCGAGTCCCAAGAGGGGAGCCGCGGGCGCCGTGGGTGGAGCA
**-13 nt** TCGACAGCGACGCCGCGAG-------------CCGCGGGCGCCGTGGGTGGAGCA

(B)

*HLA-A* **locus** **GTTYGACAGCGACGCCGCGAGCC AGAGGATGGAGCCGCGGGCGCCGTG**
**-13 nt** GTTTGACAGCGACGCCGCGAGCC--------------GCGGGCGCCGTG
**+1 nt** GTTCGACAGCGACGCCGCGAGCCCAGAGGATGGAGCCGCGGGCGCCGTG

*HLA-B* **locus** **GGGCGCCTCCTCCGCGGGTATG ACCAGGACGCCTACGACGGCAAGGATTACA**
**-10 nt** GGGCGCCTCCTC--------------------------ACCAGGACGCCTACGACGGCAAGGATTACA
**-1+17 nt** GGGCGCCTCCTCCGCGGGTAT-ACCTCCTCCGCGGGTATACCAGGACGCCTACGACGGCAAGGATTACA

*HLA-C* **locus** **TCGACAGCGACGCCGCGA GTCCAAGAGGGGAGCCGCGGGCGCCGTGGGTGGAGCA**
**+1 nt** TCGACAGCGACGCCGCGAAcTgCAcGAGGGGAGtCGCGGGCGCCGTGGGTGGAGCA
**-13 nt** TCGACAGCGACGCCGC-- -----------GAGCCGCGGGCGCCGTGGGTGGAGCA

**Figure S1. DNA sequences of the target region in gene-engineered clones.**
(A) The DNA sequence of target regions in two clones that have frameshift mutations at five target sites. (B) The DNA sequence of target regions in HLA class Ia KO clone. The single base substitutions are indicated in lower case. The red hyphen indicates a deleted nucleotide. Inserted nucleotides are represented in blue. The target sequences of gRNA are underlined.

Fig S2

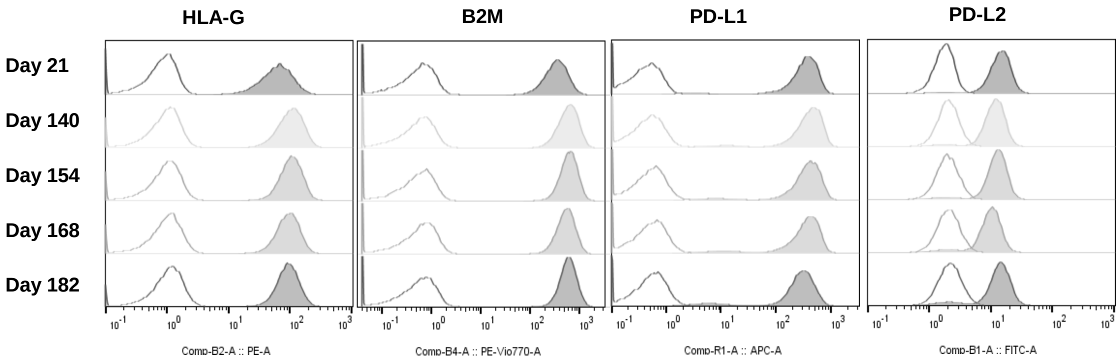


**Figure S2. The expression of transgenes in HyPSCs was maintained during long-term culture**
The expression of transgenes in HyPSCs was analyzed at days 21, 140, 154, 168, and 182 by flow cytometry using specific antibodies.

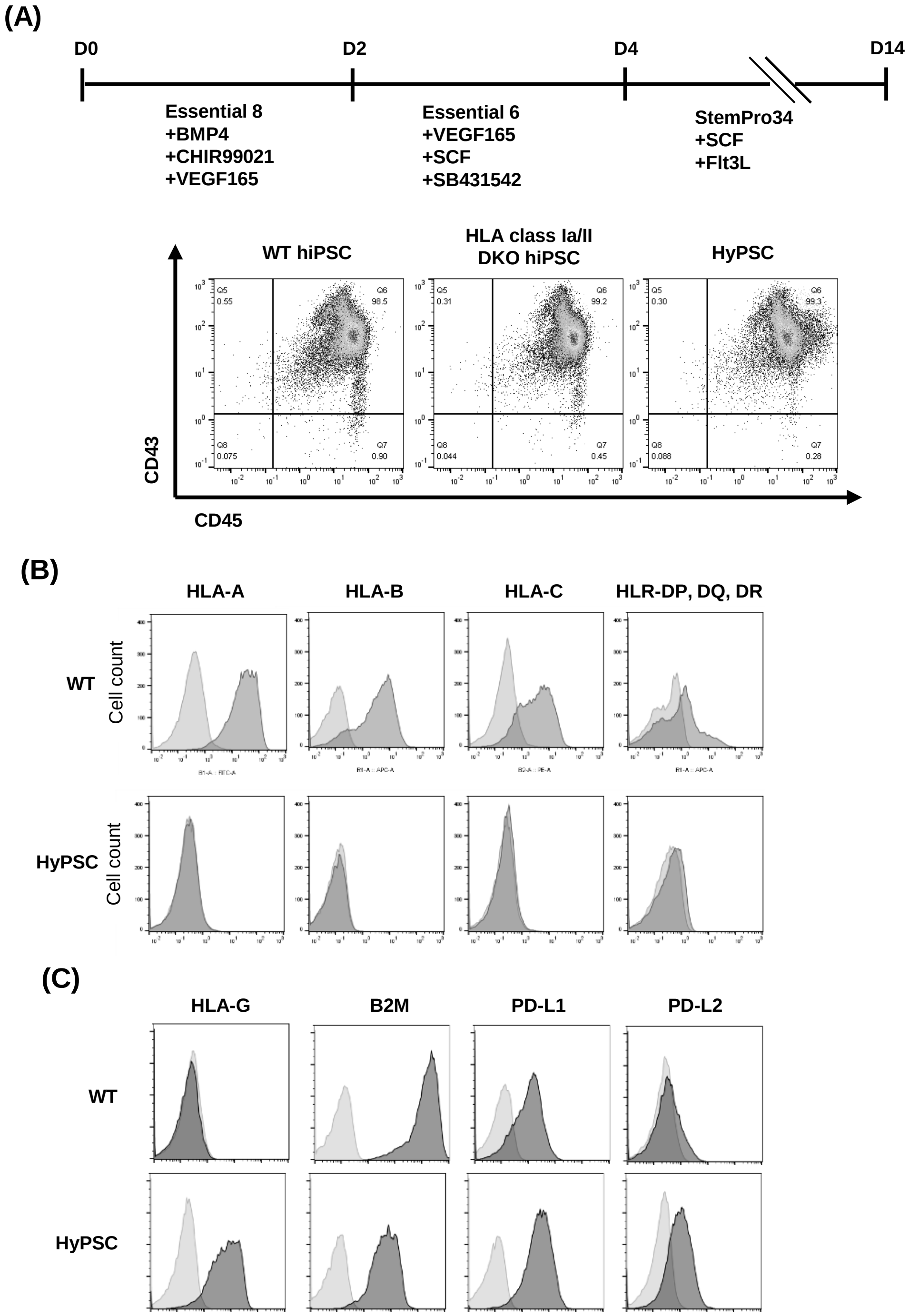


**Figure S3. Hematopoietic progenitor differentiation of HyPSCs.**
(A) The schematic presentation of the protocol for HPC differentiation (upper panel). The expression of CD45+ molecules on the surface of the engineered hiPSCs was determined by flow cytometry (lower panel). (B) The expression of HLA class Ia and II was absent from the cell surface of HyPSC-derived HPCs. (C) The expression of transgenes on HyPSC-derived HPCs was determined by flow cytometry.

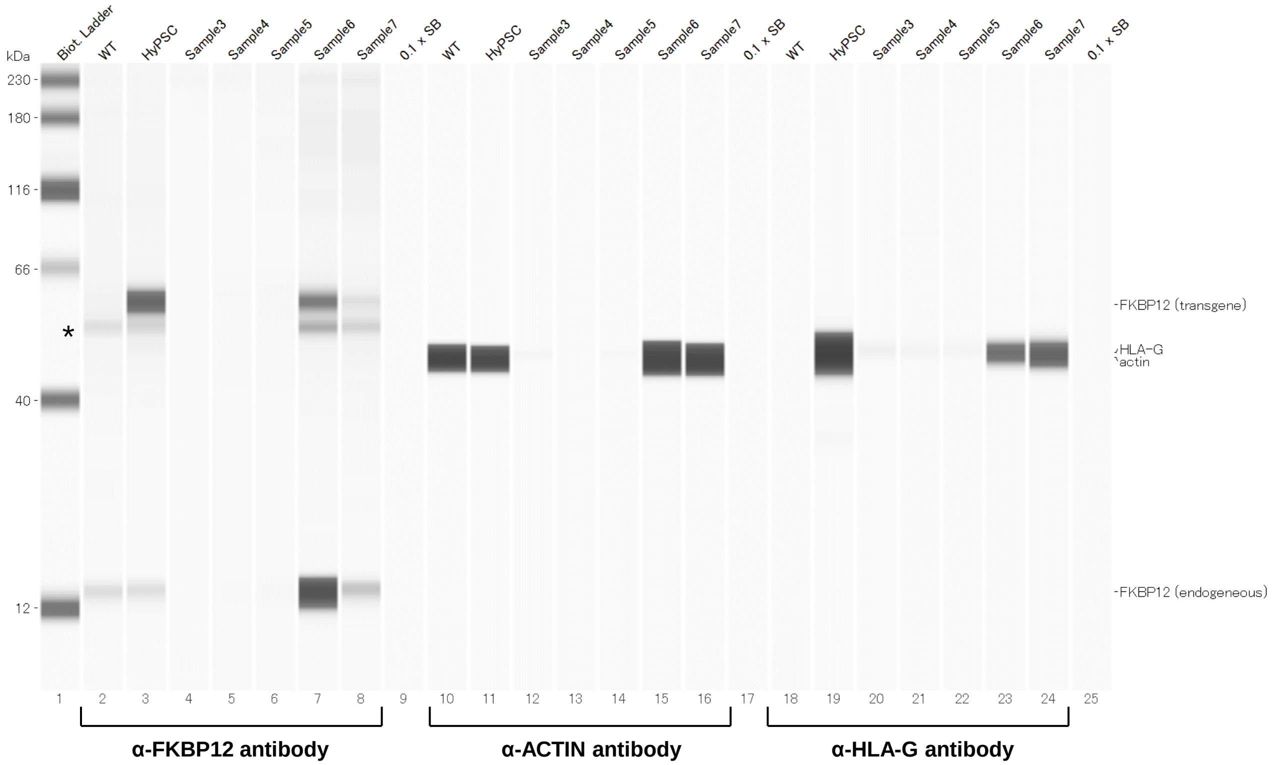


**Figure S4. A complete image of the 25 capillaries shown in Figure 2D.**
**The expression of RapaCasp9 and HLA-G proteins was detected using the Jess Automated Western Blot System (ProteinSimple). Anti-FKBP12 antibody was used to identify RapaCasp9. β-Actin was used as an internal loading control. Please note that the data from samples 3 to 7 were not included in this paper. 0.1 × SB: Sample buffer only.**
